# Biological Roles of the *O*-Methyl Phosphoramidate Capsule Modification in *Campylobacter jejuni*

**DOI:** 10.1371/journal.pone.0087051

**Published:** 2014-01-30

**Authors:** Lieke B. van Alphen, Cory Q. Wenzel, Michele R. Richards, Christopher Fodor, Roger A. Ashmus, Martin Stahl, Andrey V. Karlyshev, Brendan W. Wren, Alain Stintzi, William G. Miller, Todd L. Lowary, Christine M. Szymanski

**Affiliations:** 1 Alberta Glycomics Centre, Department of Biological Sciences, University of Alberta, Edmonton, Alberta, Canada; 2 Alberta Glycomics Centre, Department of Chemistry, University of Alberta, Edmonton, Alberta, Canada; 3 Ottawa Institute of Systems Biology, Department of Biochemistry, Microbiology, and Immunology, University of Ottawa, Ottawa, Ontario, Canada; 4 School of Life Sciences, Kingston University, London, United Kingdom; 5 London School of Hygiene and Tropical Medicine, London, United Kingdom; 6 Produce Safety and Microbiology Research Unit, Agricultural Research Service, US Department of Agriculture, Albany, California, United States of America; University of Helsinki, Finland

## Abstract

*Campylobacter jejuni* is a major cause of bacterial gastroenteritis worldwide, and the capsular polysaccharide (CPS) of this organism is required for persistence and disease. *C. jejuni* produces over 47 different capsular structures, including a unique *O*-methyl phosphoramidate (MeOPN) modification present on most *C. jejuni* isolates. Although the MeOPN structure is rare in nature it has structural similarity to some synthetic pesticides. In this study, we have demonstrated, by whole genome comparisons and high resolution magic angle spinning NMR, that MeOPN modifications are common to several *Campylobacter* species. Using MeOPN biosynthesis and transferase mutants generated in *C. jejuni* strain 81–176, we observed that loss of MeOPN from the cell surface correlated with increased invasion of Caco-2 epithelial cells and reduced resistance to killing by human serum. In *C. jejuni*, the observed serum mediated killing was determined to result primarily from activation of the classical complement pathway. The *C. jejuni* MeOPN transferase mutant showed similar levels of colonization relative to the wild-type in chickens, but showed a five-fold drop in colonization when co-infected with the wild-type in piglets. In *Galleria mellonella* waxmoth larvae, the MeOPN transferase mutant was able to kill the insects at wild-type levels. Furthermore, injection of the larvae with MeOPN-linked monosaccharides or CPS purified from the wild-type strain did not result in larval killing, indicating that MeOPN does not have inherent insecticidal activity.

## Introduction

*Campylobacter jejuni* is a Gram-negative bacterium that is a leading cause of bacterial gastroenteritis worldwide [1]. The handling and consumption of contaminated poultry are the most common routes of infection in developed countries [2], although contaminated water and dairy products can also be significant sources [3]. In developing countries, campylobacteriosis is endemic and represents a major cause of diarrheal disease and infant mortality. Symptoms of infection typically range from mild to severe inflammatory diarrhea, but in rare cases infection leads to the life-threatening autoimmune disorder known as the Guillain-Barré syndrome [4].

Capsular polysaccharide (CPS) forms the outermost structure on most bacteria and plays key roles in the interaction between the organism, host, and environment. In the case of *C. jejuni*, CPS is the major determinant of the Penner serotyping scheme [5] and has been demonstrated to be important for both serum resistance and invasion of epithelial cells [6]. The CPS produced by *C. jejuni* strains are structurally complex and highly variable, due in part to the addition of phase-variable modifications such as *O*-methyl, ethanolamine, aminoglycerol, and *O*-methyl phosphoramidate (MeOPN) groups [7], [8], [9]. Although phosphoramidates are rare in nature, our previous studies identified the presence of MeOPN modifications in ∼70% of *C. jejuni* isolates [10]. In *C. jejuni* strain 11168H, we identified four genes required for the biosynthesis of MeOPN, *cj1415−cj1418,* and two phase variable genes, *cj1421* and *cj1422,* that encode transferases responsible for the addition of MeOPN to C-3 of β-D-Gal*f*NAc or to C-4 of D-*glycero*-α-L-*gluco*-Hep, respectively [10].

While it is unknown whether MeOPN modifications are present in other *Campylobacteraceae*, their commonality among *C. jejuni* strains suggests an important biological role for these surface-expressed modifications within the species, and we have recently demonstrated that MeOPN is a receptor for several *C. jejuni* lytic bacteriophages [11], [12]. MeOPN has structural similarity to some synthetic pesticides [13], and infection studies with *C. jejuni* 11168H in the *Galleria mellonella* model showed a substantial decrease in insecticidal activity for a MeOPN biosynthesis mutant relative to the wild-type strain [14]. More recently, Maue *et al*. [15] demonstrated that disruption of a MeOPN biosynthetic gene in the virulent *C. jejuni* 81–176 strain correlated to reductions in both serum resistance and colonization in a mouse intestinal model, relative to the wild-type strain. In combination, these studies indicate that, when present, MeOPN biosynthesis is important for *C. jejuni* cellular interactions and infection.

In this study, we have determined that MeOPN modifications are prevalent among several *Campylobacter* species, and not limited to *C. jejuni*. We have mutated the MeOPN transferase gene homologues in *C. jejuni* strains 81–176 and 11168H. Both mutants exhibited similar levels of insecticidal activity compared to wild-type in the *G. mellonella* infection model, and no insecticidal activity was observed when *G. mellonella* was injected with either purified CPS or synthesized compounds containing MeOPN. However, the MeOPN mutant exhibits enhanced invasion of Caco-2 cells and reduced resistance to serum, primarily due to activation of the classical complement pathway. The mutant shows no difference in colonization of chickens compared to the wild-type, but the mutant shows a drop in colonization in piglets in co-infection studies with the wild-type. Our data suggests that, when present, MeOPN has a contributory role in pathogenesis.

## Materials and Methods

### Reagents, Strains, Plasmids and Growth Conditions

Unless otherwise specified, all reagents were obtained from Sigma-Aldrich Canada (Oakville, ON), and all media were obtained from Difco Laboratories (Detroit, MI). Restriction enzymes were obtained from New England Biolabs (Herts, UK) and T4 DNA ligase was obtained from Promega (Southampton, UK). All *Campylobacter* strains (Table 1) were grown under microaerobic conditions (10% CO_2_, 5% O_2_, 85% N_2_) at 37°C. For NMR experiments, *Campylobacter* strains were grown on Brain Heart Infusion agar supplemented with 5% (v/v) defibrinated horse blood. Otherwise, *C. jejuni* 81–176 wild-type and mutant strains were routinely propagated on Mueller-Hinton (MH) agar or in MH broth with agitation at 100 r.p.m. Where appropriate, media were supplemented with 30 µg ml^−1^ kanamycin (Km) and 20 µg ml^−1^ chloramphenicol (Cm).

**Table 1:** Bacterial strains.

Strain		Description	Ref/Source
*Campylobacter avium* LMG 24591^T^			[28]
*Campylobacter coli* RM2228			[29]
*Campylobacter concisus* RH 13826.98			[30]
*Campylobacter cuniculorum* LMG 24588^T^ (RM8641)			[31]
*Campylobacter curvus* 525.92		Human oral isolate	Al Lastovica
*Campylobacter fetus* subsp. *fetus* 82–40			[32]
*Campylobacter fetus* subsp. *venerealis* 97/608			[33]
*Campylobacter gracilis* ATCC 33236^T^			[34]
*Campylobacter helveticus* CCUG 30566 (RM4087)		Feline	Stephen On
*Campylobacter hominis* ATCC BAA-381^T^			[35]
*Campylobacter hyointestinalis* subsp. *hyointestinalis* LMG 9260		Human clinical	Stephen On
*Campylobacter hyointestinalis* subsp. *lawsonii* CCUG 27631		Porcine	Stephen On
*Campylobacter insulaenigrae* NCTC 12927^T^ (RM5435)			[36]
*Campylobacter jejuni* strains			
	11168H	Hypermotile variant	[37]
	11168H Δ*cj1421-cj1422*::*kan^r^*	MeOPN transferase mutant	[10]
	81–176		[38]
	81–176 *cjj81176_1420*::*kan^r^*/*cjj81176_1435*::*cam^r^*	MeOPN transferase mutant	This study
	81–176 *kpsM*::*kan^r^*	acapsular mutant	[6]
	NCTC 11168		[39]
*Campylobacter lanienae* NCTC13004^T^			[40]
*Campylobacter lari* subsp. *concheus* LMG 11760 (RM2825)			[41]
*Campylobacter lari* subsp. *lari* RM2100			[29]
*Campylobacter lari* (UPTC) NCTC 11845 (RM3659)			[42]
*Campylobacter mucosalis* CCUG 21559			[43]
*Campylobacter peloridis* LMG 11251			[41]
*Campylobacter rectus* ATCC 33238^T^			[34]
*Campylobacter showae* ATCC 51146^T^			[44]
*Campylobacter sputorum* bv. fecalis CCUG 20703			[45]
*Campylobacter sputorum* bv. paraureolyticus LMG 11764			[45]
*Campylobacter sputorum* bv. sputorum RM3237		Unknown	Unknown
*Campylobacter subantarcticus* LMG 24377^T^ (RM8523)			[46]
*Campylobacter upsaliensis* RM3195			[29]
*Campylobacter upsaliensis* RM3940			[47]
*Campylobacter ureolyticus* RIGS9880		Human clinical	Stephen On
*Campylobacter volucris* LMG 24379			[46]

T type strain.

### *Campylobacter* Speciation/screening for Orthologues of *cj1416–1418*

Thirty-one proteomes, predicted from the complete or draft genomes of all current, validly-described *Campylobacter* taxa (Table 1), were combined into a FASTA-formatted file, which was then used to construct the binary files for a BLASTP database. This database was queried using BLASTP and the protein sequences of *C. jejuni* strain NCTC 11168 loci Cj1416-Cj1418. Positive matches were scored as those matches with >35% similarity and an alignment length across either the query or match sequences of >75%.

### High Resolution Magic Angle Spinning (HR-MAS) NMR Spectroscopic Analysis of *Campylobacter* Strains

The methods were followed as previously described [7], with minor modifications. Except where noted, all HR-MAS spectra were acquired in D_2_O on a 600 MHz spectrometer, equipped with a ^1^H{^15^N–^31^P} 4 mm pulsed field gradient (PFG) indirect-detection nanoprobe. The first increment of ^1^H–^31^P heteronuclear signal quantum correlation spectra (1D HSQC spectra) were acquired with the transients noted: 1780 for *C. insulaenigrae* RM5435; 2048 for *C. lari* UPTC NCTC 11845; 12,288 for *C. lari* subsp. *concheus* LMG 11760; 1320 for *C. subantarcticus* RM8523; 1532 for *C. cuniculorum* LMG 24588; 3072 for *C. upsaliensis* RM3940; 512 for *C. upsaliensis* RM3195, *C. helveticus* CCUG 30566, *C. jejuni* NCTC 11168, and the *C. jejuni* 81–176 *cjj81176_1415* mutant; 256 for *C. jejuni* 81–176 and the *C. jejuni* 81–176 *cjj81176_1420*/*cjj81176_1435* mutant. Note that the spectrum for the *C. jejuni* 81–176 *cjj81176_1415* mutant was obtained on a 500 MHz spectrometer.

### Construction of a MeOPN Transferase Knockout Mutant in *C. jejuni* 81–176

The *cjj81176_1420* and *cjj81176_1435* genes of *C. jejuni* 81–176 (Genome accession number: NC_008787) were disrupted by insertional inactivation and allelic replacement. Briefly, *cjj81176_1420* and *cjj81176_1435 *were PCR amplified using the primer pairs ak265/ak266 and ak265/267 with the following sequences: ak265∶5′-GCTCTAGAAGGAGTTTAAAATGTATAACCCAAACTCAGCTATAGAAAGAG-3′; ak266∶5′-GCTCTAGATTACAAATCTTTTTCCTGAATATCACCATCCAAC-3′; ak267∶5′-GCTCTAGACTATGTTTTAATTTCTTTATAACTATACCAATTTTTAC-3′. The 1.8 kb PCR products were cloned into the pGEM-T Easy vector (Promega). After confirmation of the inserts via restriction analysis, the recombinant plasmids were used for construction of *kan^r^* and *cam^r^* derivatives for insertional inactivation of genes *cjj81176_1420* and *cjj81176_1435*, respectively. The blunt-ended BamHI fragment of pJMK30 (sourse of the *kan^r^* cassette) was inserted into a unique HindIII site (after blunt-ending) of *cjj81176_1420*, whilst the *cam^r^* cassette-carrying the SmaI fragment of plasmid pAV35 was inserted into a unique SwaI site of gene *cjj81176_1435* [16]. Orientations of the *cam^r^* and *kan^r^* cassettes were verified using restriction analysis to make sure that the antibiotic resistance genes are in colinear orientation with the target genes. The latter is essential for prevention of a negative polar effect.

After transformation of the *cjj81176_1420*::*kan^r^* derivative into 81–176 and selection of colonies on a Km plate (50 µg ml^−1^) the products of recombination were confirmed by PCR using primers ak265/ak266. The derived 81–176 *cjj81176_1420*::*kan^r^* mutant was then used for insertional inactivation of gene *cjj81176_1435* via trasformation with the *cjj81176_1435*::*cam^r^* construct and selection of recombinant clones on plates supplemented with both Km (50 µg ml^−1^) and Cm (10 µg ml^−1^). The resultant *cjj81176_1420*::*kan^r^*/*cjj81176_1435*::*cam^r^* MeOPN transferase double mutant isolates were verified by PCR with ak265/266, ak265/ak267 and ak265 with ak237 (reverse primer within the *can^r^* cassette; 5′-TCCTGAACTCTTCATGTCGATTG-3′) producing products of 3.3 kb, 2.7 kb and 1.4 kb respectively as expected. Several independent confirmed clonal isolates were stored in 50% glycerol at −80°C.

### Construction of a MeOPN Biosynthesis Knockout Mutant in *C. jejuni* 81–176

The *cjj81176_1415 *gene of *C. jejuni* 81–176 was disrupted by insertional inactivation and allelic replacement. Briefly, a mutagenesis construct was generated by PCR amplification of the disrupted *cj1416-kan^r^* gene from the 11168H *cj1416* mutant we used previously [10] (and also used by Champion *et al*. [14]) using primers CS-350 (5′- CACTAAATCAGCCTCTGGTTTATC-3′) and CS-351 (5′- AAAAGAAGATTTGGCTCATCTTG-3′), followed by ligation of the 3.2 kb PCR product into the pGEM-T Easy vector (Promega). After natural transformation of the *cjj81176_1415*::*kan^r^* derivative into 81–176 and selection of colonies on a BHI-Blood Km agar plate (25 µg ml^−1 ^Km), 81–176 *cjj81176_1415*::*kan^r^* mutant isolates were verified by PCR using the CS-350/CS-351 primer pair, producing a 3.2 kb product as expected.

### Reverse Transcriptase PCR

Reverse transcriptase PCR experiments were performed with wildtype and mutant strains to examine whether transcript levels of genes located immediately downstream from insertionally disrupted genes were affected in the MeOPN biosynthesis and MeOPN transferase knockout mutant strains used in this study (Fig. S1). Total RNA extracts were obtained from *C. jejuni* 11168H and 81–176 wildtype, 11168H *cj1420*::*kan^r^* mutant, 11168H *cj1421*/*cj1422*::*kan^r^* mutant, 81–176 *cjj81176_1415*::*kan^r^* mutant, and 81–176 *cjj81176_1420*::*kan^r^*/*cjj81176_1435*::*cam^r^* mutant strains using the Aurum™ Total RNA Mini Kit (Bio Rad Laboratories, Inc., Hercules, CA). First strand synthesis of cDNA from RNA preparations was performed using the SuperScript III First-Strand Synthesis system (Life Technologies, Carlsbad, CA), using the following specific primers: CS-645 (5′- CATTAGCTCGAGGTTTTTTTGATATTG-3′) for *cjj81176_1414* and *cj1415*; CS-1247 (5′- GGAATCTCTATACTTTGCATATG-3′) for *cjj81176_1419* and *cj1420*; CS-1249 (5′- GGACACTAACTCAAATGGTAG-3′) for *cjj81176_1434*; CS-869 (5′- ATGACTTGACGTCGTCCACACCTT -3′) for 16S rRNA as an internal control. PCR reactions were performed using resultant cDNA preparations with Taq polymerase (Life Technologies) and the following primer pairs: CS-644 (5′- GAGGTGCCATATGAAAAATAATCC-3′) and CS-645 for *cjj81176_1414* and *cj1415*; CS-1246 (5′- CAATAGACCTGCTTATAGTCC-3′) and CS-1247 for *cjj81176_1419* and *cj1420*; CS-1248 (5′- GTAAAATACGTGGCTGTTGTC-3′) and CS-1249 for *cjj81176_1434*; CS-868 (5′- GCGCAACCCACGTATTTAGTTGCT -3′) and CS-869 for 16S rRNA as an internal control. For each PCR reaction, a 75-µl reaction mix was generated and aliquoted into 7 individual 10-µl aliquots. These aliquots were placed in a thermocycler and subjected to PCR, with individual aliquots being removed in 5-cylce increments from 5 to 35 cycles, and analyzed by agarose gel electrophoresis.

### Adherence and Invasion Assays

The human Caco-2 epithelial cell line was obtained from the American Type Culture Collection (ATCC HTB-37) and was routinely grown in Dulbecco’s minimal essential medium (DMEM) supplemented with 10% (v/v) fetal bovine serum, 2 mM L-glutamine and 1.0 mM sodium pyruvate and incubated at 37°C in a humidified atmosphere with 5% CO_2_. Two days prior to infection, cells were trypsinized and seeded into a 24-well plate at 2.5×10^5^ cells ml^−1^, according to the method of Oelschlaeger et al. [17]. On the day of infection, Caco-2 cell medium was replaced with infection medium (modified Eagle’s medium supplemented with 2 mM L-glutamine, 1 mM sodium pyruvate, and 1% fetal bovine serum) 1 h prior to addition of the bacteria. Bacterial cells from an overnight culture were harvested by centrifugation (3,000×*g*, 10 min, 20°C), washed with phosphate buffered saline (PBS), and added to Caco-2 cells to a multiplicity of infection of 100. After a 3 h incubation at 37°C in 5% CO_2_, Caco-2 cells were rinsed with 3×1 ml of PBS, lysed with 250 µl of 0.1% Triton X-100 in PBS (15 min, 20°C), and dilutions of each well were plated on MH agar to yield combined adherence and invasion numbers. Invasion levels were determined after a 3 h incubation in 0.5 ml of medium containing 250 µg ml^−1^ of gentamicin (Gm), then extensive washing prior to lysis with Triton X-100. Adherence values were determined by subtraction of the invasion values from the combined adherence and invasion values. Experiments were performed in duplicate and the averages (mean ± SEM) of three separate experiments are presented, and significance was assessed by an unpaired t-test.

### Motility Assays

To assess the motility of the *C. jejuni* 81–176 *cjj81176_1415*::*kan^r^*, *cjj81176_1420*::*kan^r^*/*cjj81176_1435*::*cam^r^* and *kpsM*::*kan^r^* mutants relative to the wild-type strain, sterile needles were dipped into liquid cultures of each strain and stabbed into semi-solid medium (thioglycollate medium containing 0.4% agar). Swarming was assessed after incubation under microaerobic conditions at 37°C for 24 h.

### Serum Resistance Assays

Serum resistance assays were performed according to the method of Blaser *et. al*. [18] with modifications. Briefly, *C. jejuni* strains were grown for 16 h in biphasic MH medium, then washed and resuspended in HEPES buffer (10 mM HEPES, 150 mM NaCl, 5 mM CaCl_2_, 5 mM KCl, 5 mM MgCl_2_, 5 mM dextrose, pH 7.4) to a final concentration of 10^6 ^CFU ml^−1^. For each culture, 100 µl aliquots of cell suspension were combined with 350 µl HEPES buffer and 50 µl of either fresh pooled normal human complement serum (NHS) (Innovative Research, Inc, Novi, MI) or heat-inactivated NHS (56°C for 45 min) (final serum concentration of 10%). Aliquots were taken at 0 min, 30 min, and 60 min, and bacterial counts were enumerated via serial dilution on MH agar. Bacterial survival in active serum was calculated as percentage of survival in inactive serum. Experiments were performed in triplicate and the averages (mean ± SEM) of three separate experiments are presented, and significance was assessed by an unpaired t-test.

To determine if the classical complement activation pathway was involved in the observed killing, serum resistance assays were performed as above in both the presence and absence of 50 mM EGTA and incubated as above for 60 min before serial dilution and plating on MH to enumerate surviving bacteria. Experiments were performed in triplicate and the averages (mean ± SEM) of three separate experiments are presented, and significance was assessed by an unpaired t-test.

### Chicken Colonization

Animal procedures were approved by the Biosciences Animal Care and Use Committee at the University of Alberta. The animals were maintained and used in accordance with the recommendations of the Canadian Council on Animal Care. Chickens were obtained from the Poultry Research Facility, Department of Agriculture, Food and Nutrition Science, University of Alberta and separated into three groups of eight birds. *C. jejuni* strains were grown overnight on MH agar with the appropriate antibiotic. Bacteria were harvested in PBS and adjusted to 10^7^ CFU/ml. One-day-old chicks were infected by oral gavage with 10^6 ^CFU of *C. jejuni* 81–176 wild-type and the *cjj81176_1420*::*kan^r^*/*cjj81176_1435*::*cam^r^* mutant in 100 µl of PBS or with PBS alone. Chicks were euthanized 6 days post-infection and the caecal content was aseptically removed, serially diluted in PBS, and plated onto Karmali selective agar plates. Colonies were counted after 2 days of incubation under microaerobic conditions (10% CO_2_, 5% O_2_, 85% N_2_) at 37°C to determine colonization levels.

### Pig Colonization

Animal procedures were approved by the Animal Care and Use Committee at the University of Ottawa. The animals were maintained and used in accordance with the recommendations of the Canadian Council on Animal Care. Five male, specific pathogen free neonatal colostrum-deprived piglets were acquired from the Canadian Food Inspection Agency and were allowed to acclimatize for at least 24 h to their environment. Overnight cultures of *C. jejuni* 81–176 wild-type and the *cjj81176_1420*::*kan^r^*/*cjj81176_1435*::*cam^r^* mutant were adjusted to an OD_600 nm_ of 1.0, combined to create a 1∶1 ratio of mutant to wild-type, and were diluted in fresh MH broth to a 10 ml culture of approximately 10^7 ^CFU/ml and used to inoculate each piglet. To confirm the exact ratio of wild-type to mutant, samples of the inoculum were serially diluted, plated on MH agar with and without Cm and enumerated.

The piglets developed a severe infection and were euthanized within 48 h post-infection. Samples were taken from the duodenum, jejunum, ileum, cecum, and colon, were homogenized, serially diluted in MH broth and plated onto Campylobacter agar base (Oxoid CM935) containing Campylobacter-selective Karmali supplements (Oxoid SR167E) with and without a 10 µg ml^−1^ kanamycin supplement for the selection of the *cjj81176_1420*::*kan^r^*/*cjj81176_1435*::*cam^r^* mutant. The plates were incubated at 37°C, under microaerobic conditions for 48 h and *C. jejuni* colonies were counted to determine colonization levels. Statistical significance was determined using a Mann−Whitney test comparing the measured wild-type to mutant ratio of the inoculum, to the ratio recovered from the piglet intestine following infection (p = 0.0005).

### *Galleria Mellonella* Killing Assays

*G. mellonella* larvae (Bug Order Inc., Morinville, AB) were stored at 4°C in woodchips until needed, and used within 7 days of being received. Overnight cultures of *C. jejuni* (grown on MH agar) were harvested and resuspended in 10 mM MgSO_4_ to a final OD_550 nm_ = 1.0 (∼1.0×10^9^ cfu ml^−1^). Stock solutions (10 mg ml^−1^) of synthesized MeOPN-linked monosaccharides (see below) (Ashmus et al., in preparation) and 1 mg ml^−1^ CPS were prepared immediately prior to injection. After swabbing larvae with ethanol, 5 µl aliquots of cell suspension or diluted compound were injected into the left hindmost proleg using a 10 µl Hamilton syringe. To prevent cross-contamination, the syringe was rinsed sequentially with methanol then water between groups. After injection, larvae were incubated at 37°C for 24 h, and survival numbers recorded. Larvae that were not responsive to touch were scored as dead. *Campylobacter* injection results represent the mean (±SEM) of five independent experiments, MeOPN-containing compound injection results represent the mean (±SEM) of four independent experiments, and the 81–176 CPS injection data was the result of a single experiment. In all cases, each individual experiment consisted of three technical replicates, each containing 10 larvae.

### Isolation of Capsular Polysaccharide

The CPS of *C. jejuni* 81–176 was purified according the enzymatic isolation method of McNally et al. [8] to avoid the partial loss of MeOPN modifications observed with phenol-based methods.

### Synthesis of MeOPN-linked Monosaccharides

The synthesis of methyl 2-*O*-(methylphosphoramidyl)-α-D-glucopyranoside (2-MeOPN-Glc), methyl 6-*O*-(methylphosphoramidyl)-α-D-glucopyranoside (6-MeOPN-Glc), methyl 2-*O*-(methylphosphoramidyl)-α-D-galactopyranoside (2-MeOPN-Gal), and methyl 6-*O*-(methylphosphoramidyl)-α-D-galactopyranoside (6-MeOPN-Glc) will be described in a separate publication (unpublished data).

## Results

### MeOPN Biosynthetic Genes are Prevalent Amongst *Campylobacter* Species

**Figure 1:**
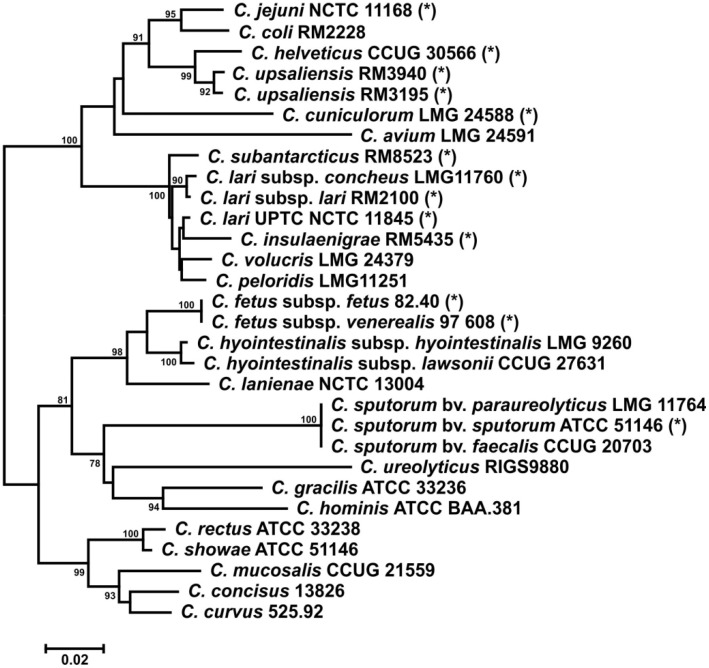
Prevalence of MeOPN biosynthesis genes within the *Campylobacter* genus. Dendrogram of AtpA amino acid sequences of representative *Campylobacter* species, with strains containing orthologues of the MeOPN biosynthesis genes (*cj1416-1418*) indicated with an asterisk. The scale bar represents substitutions per site. Bootstrap values of >75%, generated from 1000 replicates using the Neighbor-Joining algorithm, are shown in the nodes. The Cj1416-Cj1418 orthologues for *C. jejuni, C. lari* and *C. fetus* are present in the NCBI database: the accession number for *C. jejuni subsp. jejuni* strain NCTC 11168 is AL111168; the accession number for *C. coli* strain 76339 (genes BN865_14280-14300) is HG326877; the accession number for *C. lari subsp. lari* strain RM2100 (genes Cla_0314-316) is CP000932; the accession number for *C. fetus subsp. fetus* strain 82–40 (genes CFF8240_1630-1632) is CP000487; and the accession number for *C. fetus subsp. venerealis* strain NCTC 10354 (genes CFV354_1762-1764) is CM001228. The accession numbers for the *atp* sequences and new Cj1416-Cj1418 orthologues from the other *Campylobacter* species are listed in Table S1.

Genetic analyses of representative members of the *Campylobacter* genus resulted in the identification of orthologues to the *C. jejuni* MeOPN biosynthesis genes *cj1416–1418* in 13 of the 30 *Campylobacter* strains analyzed (Fig. 1), with a high prevalence in strains closely related to *C. jejuni*.

### The Presence of MeOPN Biosynthetic Genes Correlates to MeOPN Production for the Majority of *Campylobacter* Species

**Figure 2:**
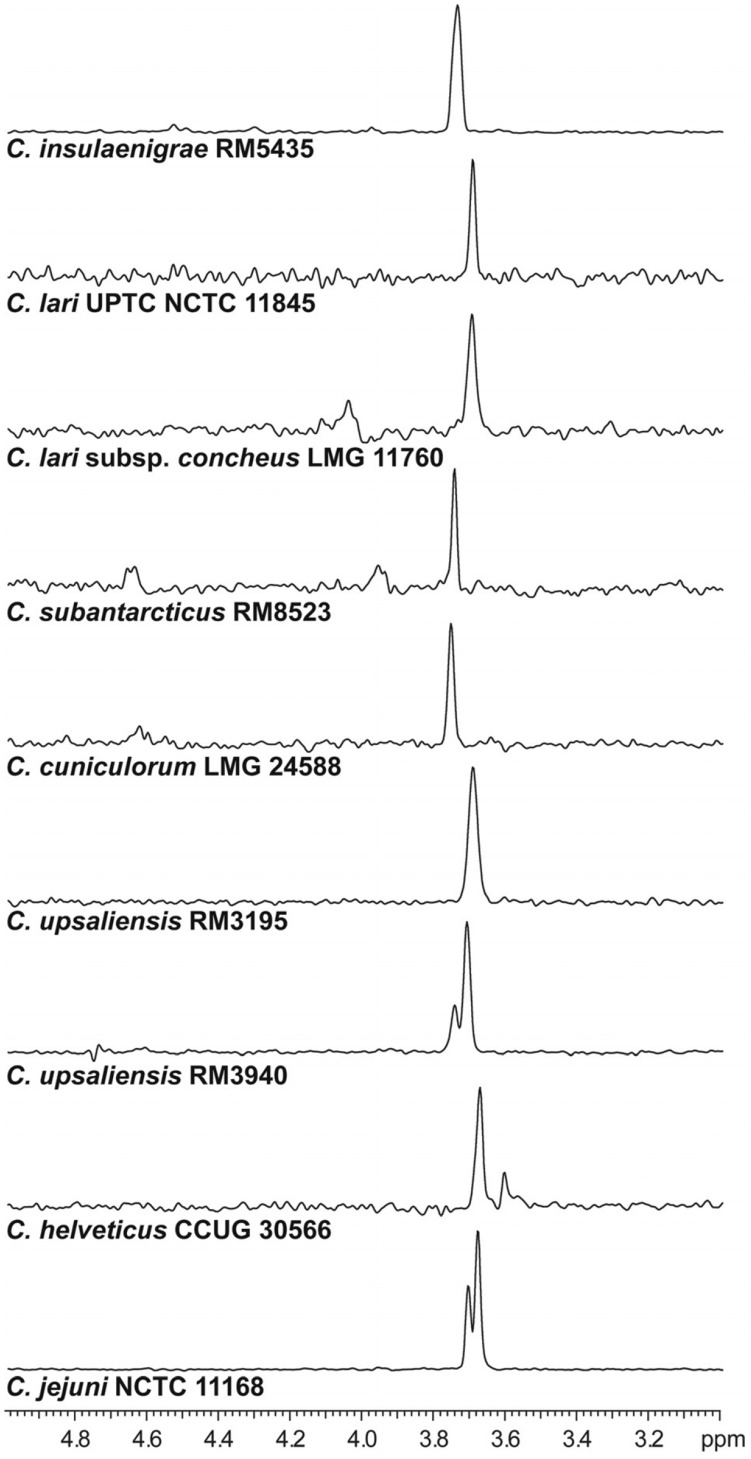
1D ^1^H–^31^P HSQC spectra of select *Campylobacter* species containing orthologues of the *cj1416*-*cj1418* genes. Spectra contain the following resonances corresponding to MeOPN: *C. insulaenigrae* RM5435, peak at 3.74 ppm; *C. lari* UPTC NCTC 11845, peak at 3.70 ppm; *C. lari* subsp. *concheus* LMG 11760, peak at 3.70 ppm; *C. subantarcticus* RM8523, peak at 3.75 ppm; *C. cuniculorum* LMG 24588, peak at 3.76 ppm; *C. upsaliensis* RM3195, peak at 3.70 ppm; *C. upsaliensis* RM3940, peaks at 3.75 and 3.71 ppm; *C. helveticus* CCUG 30566, peak at 3.68 and 3.61 ppm; *C. jejuni* NCTC 11168, peaks at 3.71 and 3.68 ppm.

A selection of the *Campylobacter* strains identified as containing orthologues of *cj1416-cj1418* were analyzed for the presence of MeOPN by HR-MAS NMR, and resonances consistent with the *O*-methyl group of MeOPN were observed in HSQC spectra of 9 of the 13 strains examined (Fig. 2). Signals corresponding to MeOPN were not observed in *C. fetus* subsp. *fetus* 82.40, *C. fetus* subsp. *venerealis* 97 608, *C. lari* subsp. *lari* RM2100, and *C. sputorum sputorum* RM3237 (data not shown).

### *C. jejuni* 81–176 Contains a Functional MeOPN Biosynthesis Locus with Two Phase Variable Orthologues of *cj1422*

**Figure 3:**
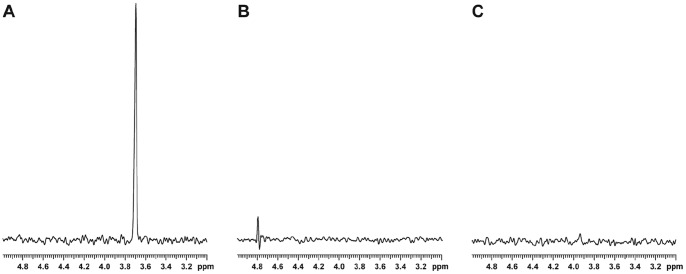
1D ^1^H–^31^P HSQC analyses of *C. jejuni* 81-176 wild-type and the MeOPN biosynthesis and transferase mutants. Intact whole cells of *C. jejuni* were analysed by HR-MAS NMR after 48 h growth on MH agar. Depicted are the 1D ^1^H–^31^P HSQC spectra, which specifically show the MeOPN resonance at 3.8 ppm in 81-176 wt (A), but not in the *cjj81176_1415*::*kan^r^* MeOPN biosynthesis mutant (B), and *cjj81176_1420*::*kan^r^/cjj81176_1435*::*cam^r^* MeOPN transferase mutant (C).

To examine the biological role of MeOPN, we used the virulent *C. jejuni* 81–176 strain. *In silico* analysis of the 81–176 genome led to the identification of a locus (*cjj81176_1414-cj81176_j1417*) with homology to the MeOPN biosynthesis locus of *C. jejuni* strain NCTC 11168 (*cj1415-cj1418*), as well as two putative MeOPN transferases (*cjj81176_1420* and *cj81176_j1435*) with homology to the *cj1422* MeOPN transferase gene from NCTC 11168. Unlike *cj1421* and *cj1422* in NCTC 11168, *cjj81176_1420* and *cjj81176_1435* are not adjacent to each other and *cjj81176_1435* is significantly downstream of the MeOPN biosynthesis locus but, similar to *cj1422*, both contain polyG:C tracts, causing them to be prone to phase variation. Sequencing results from our 81–176 wild-type strain indicated that *cjj81176_1435* is phased on while *cjj81176_1420* is phased off, but a *cjj81176_1420*::*kan^r^*/*cjj81176_1435*::*cam^r^* double knockout was nonetheless generated to avoid any risk of *cjj81176_1420* turning back on. HR-MAS NMR analyses indicated the presence of a single MeOPN in the 81–176 wild-type strain which was absent in the *cjj81176_1420*::*kan^r^*/*cjj81176_1435*::*cam^r^* MeOPN transferase mutant (Fig. 3).

### Loss of MeOPN on the Cell Surface Results in Increased Invasion of Epithelial Cells

**Figure 4:**
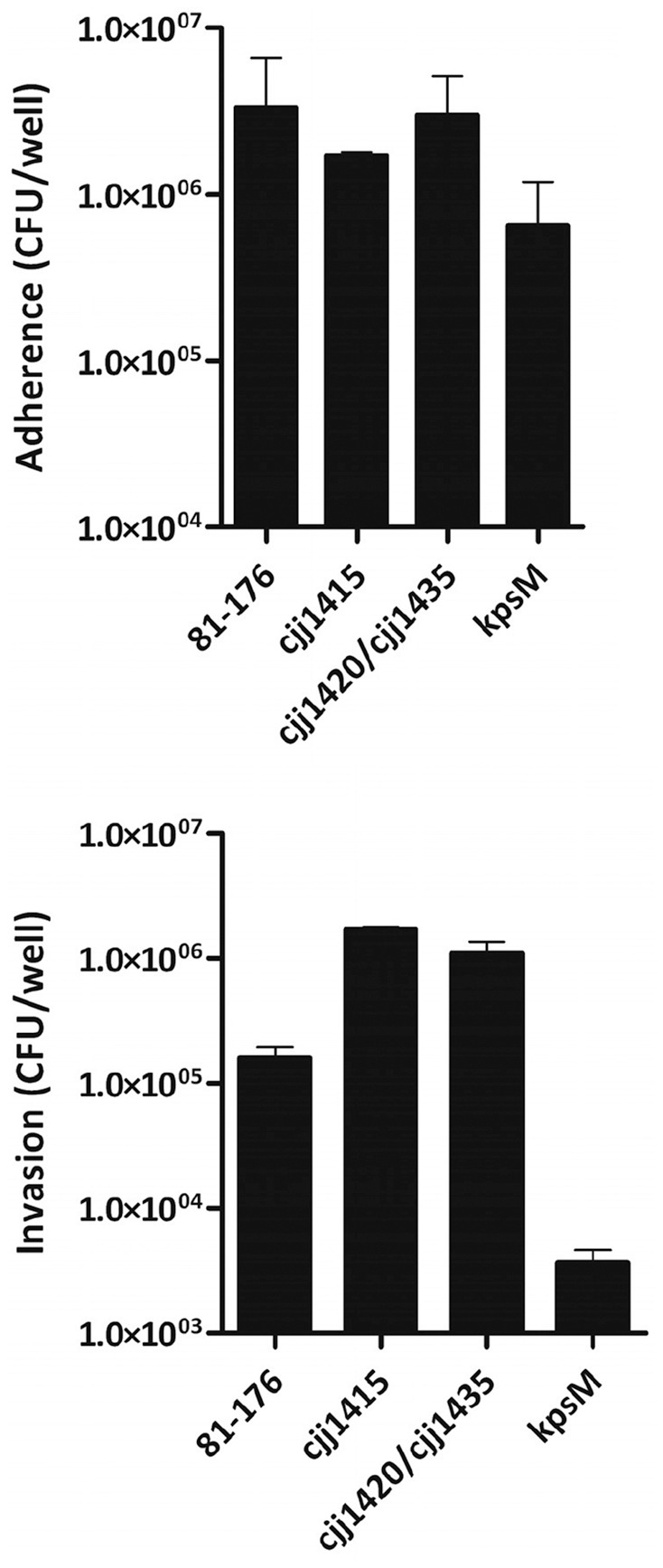
Loss of MeOPN results in increased invasion of Caco-2 cells. Adherence and invasion of Caco-2 cells by *C. jejuni* 81-176 wild-type, *cjj81176_1415*::*kan^r^* (MeOPN biosynthesis mutant), *cjj81176_1420*::*kan^r^/cjj81176_1435*::*cam^r^* (MeOPN transferase mutant), and *kpsM*::*kan^r^* (capsule mutant) in the gentamicin protection assay. Note that a significant difference (p≤0.05, unpaired t-test) in adherence was observed between *C. jejuni* 81-176 wild-type and the *kpsM*::*kan^r^* mutant, and significant differences in invasion were observed between the *C. jejuni* wild-type, and the *cjj81176_1415*::*kan^r^* (MeOPN biosynthesis mutant), *cjj81176_1420*::*kan^r^/cjj81176_1435*::*cam^r^* and *kpsM*::*kan^r^* mutants. Results represent the mean (± SEM) of three independent experiments.

To examine whether or not the presence of MeOPN on the cell surface has a significant effect on adherence and invasion, the ability of the *cjj81176_1420*::*kan^r^*/*cjj81176_1435*::*cam^r^* mutant to adhere to and invade Caco-2 cells, relative to both the wild-type 81–176 and the acapsular 81–176 *kpsM*::*kan^r^* mutant, was compared. The *cjj81176_1420*::*kan^r^*/*cjj81176_1435*::*cam^r^* mutant exhibited similar adherence levels to wild-type, while the acapsular *kpsM*::*kan^r^* mutant showed a nearly 10-fold decrease in adherence relative to the wild-type strain (Fig. 4). These results indicate that although the capsule is important for adherence, the presence of MeOPN on the capsule is not. When invasion was assessed, the *cjj81176_1420*::*kan^r^*/*cjj81176_1435*::*cam^r^* mutant displayed a nearly 10-fold increase in invasion relative to wild-type, while the control *kpsM*::*kan^r^* mutant invaded to significantly lower levels than wild-type, as expected (Fig. 4). Motility assays were performed to address the possibility that the differences observed between strains were due to changes in motility, and no significant differences in motility were observed between the three strains (data not shown). To ensure that the observed changes in invasion were due to loss of MeOPN, we also constructed a MeOPN biosynthesis mutant (*cjj81176_1415*::*kan^r^*) with homology to *cj1416* from NCTC 11168. Similar to the MeOPN transferase mutant, the biosynthesis mutant did not show a resonance for MeOPN in the HR-MAS NMR analysis (Fig. 3 B) or a difference in motility or adherence, but did show a statistically significant increase in invasion.

### Loss of MeOPN from the Bacterial Surface Leads to a Pronounced Decrease in Serum Resistance

**Figure 5:**
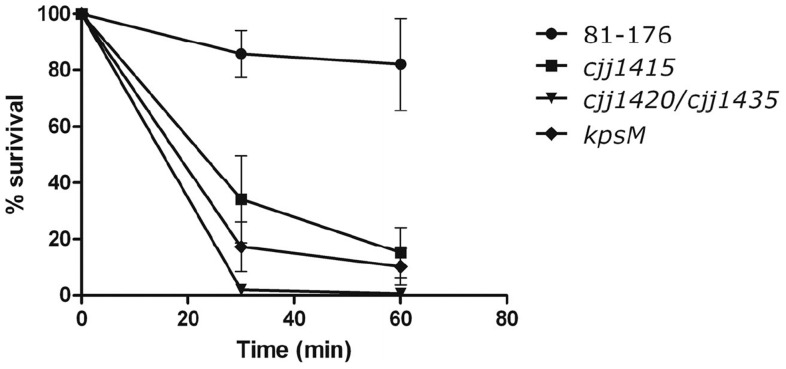
Loss of MeOPN results in a decrease in serum resistance. Survival of *C. jejuni* 81-176 wild-type, *cjj81176_1415*::*kan^r^* (MeOPN biosynthesis mutant), *cjj81176_1420*::*kan^r^/cjj81176_1435*::*cam^r^* (MeOPN transferase mutant), and *kpsM*::*kan^r^* (capsule mutant) in 10% natural human complement serum relative to survival in heat inactivated serum. Serum resistance was significantly different between *C. jejuni* 81-176 wild-type, the *cjj81176_1415*::*kan^r^* (MeOPN biosynthesis mutant), and the *cjj81176_1420*::*kan^r^/cjj81176_1435*::*cam^r^* mutant at both 30 and 60 min (p<0.0001). Serum resistance was significantly different between *C. jejuni* 81-176 wild-type and the *kpsM*::Km^R^ mutant at both 30 and 60 min (p = 0.005 and p = 0.014, respectively) using an unpaired t-test. Results represent the mean (± SEM) of three independent experiments.

Given that MeOPN on the cell surface of *C. jejuni* does not enhance, but rather impairs the invasion process, we examined whether MeOPN has any immunomodulatory effects that may be beneficial in early stages of the infection process. When incubated with normal human serum (NHS), the *cjj81176_1415*::*kan^r^* and *cjj81176_1420*::*kan^r^*/*cjj81176_1435*::*cam^r^* mutants and the acapsular *kpsM*::*kan^r^* mutant exhibited marked sensitivity to serum-mediated killing relative to wild-type (Fig. 5), indicating that the presence of MeOPN on the cell surface of *C. jejuni* provides significant protection against the humoral immune system.

### The MeOPN Modifications on 81–176 CPS have a Protective Influence Against Serum-mediated Killing via the Classical Complement Activation Pathway

**Figure 6:**
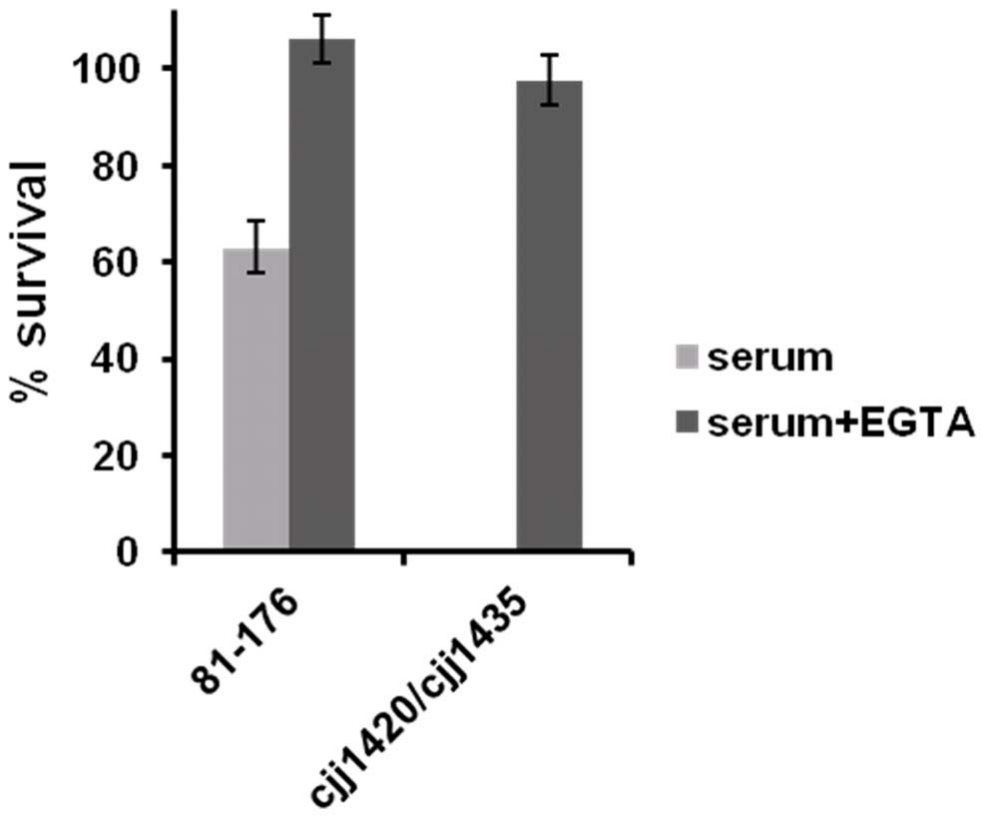
MeOPN modifications provide protection against serum mediated killing by interfering with the classical complement activation pathway. Survival of *C. jejuni* 81-176 wild-type and *cjj81176_1420*::*kan^r^/cjj81176_1435*::*cam^r^* (MeOPN transferase mutant) in 10% natural human complement serum. The samples were tested in the presence or absence of 50 mM EGTA after 60 min incubation, relative to survival in heat inactivated serum. Serum resistance was significantly different between *C. jejuni* 81-176 wild-type in the presence versus absence of EGTA (p = 0.0030), the *cjj81176_1420*::*kan^r^/cjj81176_1435*::*cam^r^* mutant in the presence versus absence of EGTA (p = 0.0014), and the 81-176 wild-type versus the *cjj81176_1420*::*kan^r^/cjj81176_1435*::*cam^r^* mutant in the absence of EGTA (p = 0.0047) using an unpaired t-test. Results represent the mean (± SEM) of three independent experiments. Note that both the wild-type and MeOPN transferase mutant approach 100% survival in the presence of EGTA.

We speculated that the pronounced serum mediated killing observed with the mutants was primarily due to the classical pathway of complement activation since the commercial serum that we used contained antibodies against *C. jejuni* (data not shown). Therefore, we examined the effects of the presence or absence of 50 mM EGTA on the level of serum resistance observed (Fig. 6). The *cjj81176_1420*::*kan^r^*/*cjj81176_1435*::*cam^r^* mutant was unable to survive a 60 min incubation in serum assays without EGTA, but ∼100% survival was observed when EGTA was included, indicating that the observed serum killing is indeed primarily due to activation of the classical complement pathway. Furthermore, the wild-type strain showed approximately 60% survival in the absence of EGTA, but ∼100% survival in the presence of EGTA, indicating that the presence of MeOPN offers partial, but not complete, protection against serum killing via the classical complement pathway.

### Chicken Colonization Levels are not Affected by the Presence or Absence of MeOPN on the Cell Surface

**Figure 7:**
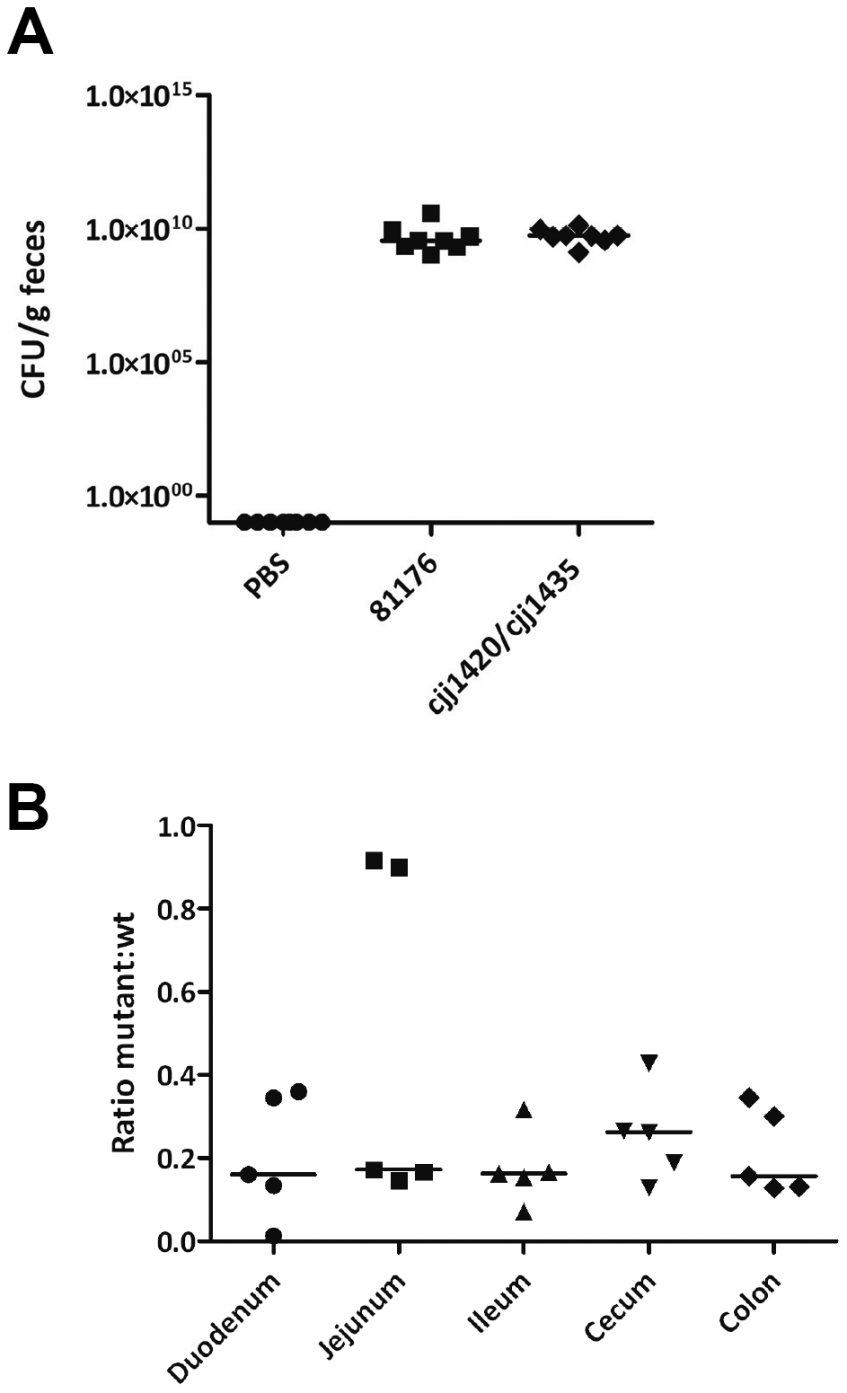
Influence of MeOPN on colonization. (**A**) Colonization levels of *C. jejuni* 81-176 wild-type and *cjj81176_1420*::*kan^r^/cjj81176_1435*::*cam^r^* (MeOPN transferase mutant) in a chicken colonization model, 6 days post-infection. (**B**) Relative colonization levels of *C. jejuni* 81-176 wild-type and the MeOPN transferase mutant in a competitive piglet infection model. Note that no significant difference was observed between strains in the chicken colonization model. In the competitive piglet infection model, the MeOPN transferase mutant displays a 5-fold reduction in colonization relative to wild-type comparing the measured wild-type-to-mutant ratio of the inoculum to the ratio recovered from the piglet intestine following infection (p = 0.0005). Statistical significance was determined using a Mann−Whitney test. Horizontal bars represent median values.

Because the presence of MeOPN on the cell surface of *C. jejuni* negatively influences invasion of human Caco-2 cells, we examined whether or not MeOPN has any effect on the ability of *C. jejuni* to colonize chickens. No significant difference in colonization levels was observed between the *cjj81176_1420*::*kan^r^*/*cjj81176_1435*::*cam^r^* mutant and the wild-type strain (Fig. 7), suggesting that MeOPN does not play an important role in chicken colonization.

### MeOPN is Contributory to Piglet Colonization

While MeOPN was not found to play a role in colonization of chickens, we examined the effects of MeOPN on colonization in piglets. Due to inherent variation in colonization levels between individual animals, competitive colonization assays were performed allowing the relative colonization efficiencies of the *cjj81176_1420*::*kan^r^*/*cjj81176_1435*::*cam^r^* and wild-type strains in an individual animal to be evaluated. The numbers of colonizing bacteria were enumerated from samples of each section of the intestine (duodenum, jejunum, ileum, cecum, and colon), and the ratio of *cjj81176_1420*::*kan^r^*/*cjj81176_1435*::*cam^r^* versus wild-type recovered was determined. The mutant was significantly outcompeted by the wild-type strain with a 5-fold reduction in colonization level (Fig. 7), indicating that the presence of MeOPN plays a contributory role in piglet colonization.

### MeOPN-containing Compounds and MeOPN-modified CPS do not have Insecticidal Activity against *G. mellonella*

**Figure 8:**
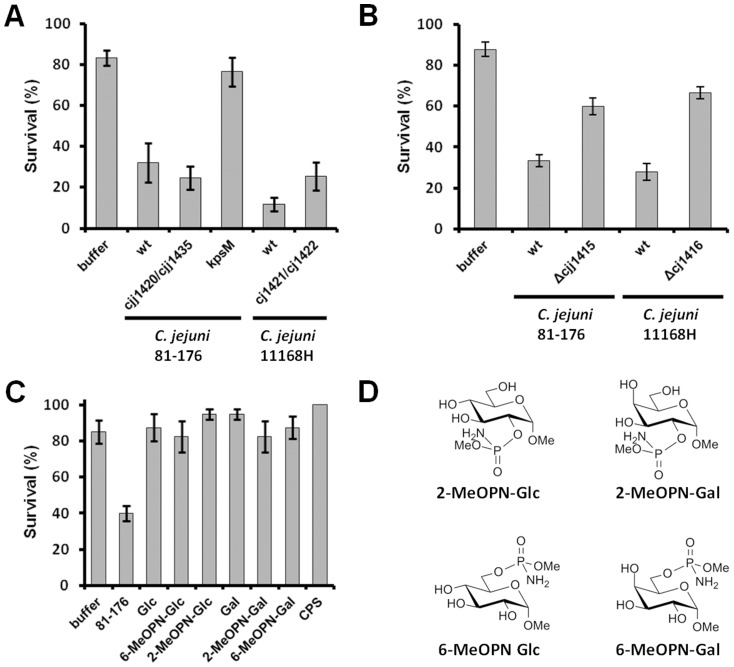
MeOPN does not have insecticidal activity against *Galleria mellonella*. (A) Insecticidal activity of *C. jejuni* 81-176 wild-type, *cjj81176_1420*::*kan^r^/cjj81176_1435*::*cam^r^* (MeOPN transferase mutant), *C. jejuni* 81-176 *kpsM*::*kan^r^* (capsule mutant), *C. jejuni* 11168H wild-type, and *C. jejuni* 11168H *cj1421-1422*::*kan^r^* (MeOPN transferase mutant). (B) Insecticidal activity of *C. jejuni* 81-176 wild-type, *cjj81176_1415*::*kan^r^* (MeOPN biosynthesis mutant), *C. jejuni* 11168H wild-type, and *C. jejuni* 11168H *cj*1416::*kan^r^* (MeOPN biosynthesis mutant). (C) Insectidal activity of *C. jejuni* 81-176 wild-type, *C. jejuni* 81-176 purified CPS, or MeOPN-containing compounds in a *G. mellonella* larval model. (D) Chemical structures of MeOPN-containing compounds. Glc – methyl α-D-glucopyranoside; 2-MeOPN-Glc - methyl 2-*O*-(methylphosphoramidyl)-α-D-glucopyranoside; 6-MeOPN-Glc - methyl 6-*O*-(methylphosphoramidyl)-α-D-glucopyranoside; Gal – methyl α-D-galactopyranoside; 2-MeOPN-Gal - methyl 2-*O*-(methylphosphoramidyl)-α-D-galactopyranoside; 6-MeOPN-Glc - methyl 6-*O*-(methylphosphoramidyl)-α-D-galactopyranoside; CPS – purified capsular polysaccharide from 81-176 wild-type. Survival percentages were significantly different between wild-type 81-176 and the *kpsM*::*kan^r^* mutant (p = 0.0031), the *cjj81176_1420*::*kan^r^/cjj81176_1435*::*cam^r^* and *kpsM*::*kan^r^* mutants (p = 0.0026), and between wild-type 81-176 and all tested MeOPN-containing compounds (p<0.005 in all cases). Note that no statistically significant difference was observed between the wild-type and MeOPN transferase mutant in either the 81-176 (p = 0.264) or 11168H (p = 0.063) strains. Results represent the mean (±SEM) of five independent experiments in (A) and four independent experiments in (C) except for 81-176 CPS, which was the result of a single experiment.

A recent study by Champion *et al*. [14] demonstrated that the *cj1416*::*kan^r^* MeOPN biosynthetic mutant in *C. jejuni* 11168H showed a marked reduction in killing of *G. mellonella* larvae compared to wild-type, and that killing was restored in a complemented mutant. These results suggested that MeOPN has insecticidal activity, consistent with the fact that MeOPN has similarity to some synthetic pesticides. We assessed the *cjj81176_1420*::*kan^r^*/*cjj81176_1435*::*cam^r^* mutant for the ability to kill *G. mellonella*, because this mutant is not deficient in MeOPN biosynthesis but is unable to transfer MeOPN to the capsule. Interestingly, the *cjj81176_1420*::*kan^r^*/*cjj81176_1435*::*cam^r^* mutant was able to kill *G. mellonella* to similar levels as wild-type while the acapsular *kpsM*::*kan^r^* mutant showed a significant decrease in killing (Fig. 8 A). To investigate the possibility that this phenomenon is unique to the *C. jejuni* 81–176 background, the abilities of wild-type *C. jejuni* 11168H and its MeOPN transferase mutant (*cj1421*-*cj1422*::*kan^r^*) [10] to kill *G. mellonella* were also assessed. In this background, the MeOPN transferase mutant in the *C. jejuni* 11168H background was also able to kill *G. mellonella* to similar levels to the wild-type, indicating that the presence of MeOPN on the CPS surface is not required for insecticidal activity for either *C. jejuni* 11168H or *C. jejuni* 81–176. In contrast, both the *C. jejuni* 81–176 *cjj81176_1415*::*kan^r^* and *C. jejuni* 11168H *cj1416*::*kan^r^* [10] MeOPN biosynthesis mutants showed reduced killing of *G. mellonella* (Fig. 8 B), consistent with Champion *et al*. [14].

Therefore, to more directly examine the insecticidal potential of MeOPN, *G. mellonella* larvae were injected with chemically-synthesized MeOPN-functionalized monosaccharides, and MeOPN-containing CPS purified from the 81–176 wild-type strain. In all cases, these compounds showed no larval killing relative to the PBS control (Fig. 8 C), indicating that the MeOPN functionality is not inherently insecticidal against *G. mellonella*.

### MeOPN Biosynthesis Mutants Show Reduced Transcription of Downstream Gene

RT-PCR experiments were done on all four MeOPN biosynthesis and transferase mutants to compare transcript levels of the downstream genes (Fig. S1). Although all downstream genes were transcribed, *cjj1414*::*kan^r^* in 81–176 and *cj1415*::*kan^r^* in 11168H showed reduced expression compared to wild-type levels.

## Discussion

Since our initial identification of MeOPN on the CPS of *C. jejuni* NCTC 11168 [7], we have identified the genes involved in the biosynthesis and transfer of this unusual modification, as well as demonstrated that this modification is prevalent among most *C. jejuni* isolates [10]. However, little is known regarding the prevalence of this modification outside of *C. jejuni* and the biological role of this modification is still not well understood. In this study we sought to determine how common this modification is among the *Campylobacter* genus and to further explore its biological importance.

Genetic analyses of representative members of the *Campylobacter* genus indicated that orthologues of the MeOPN biosynthetic genes *cj1416-cj1418* were not limited only to *C. jejuni.* Furthermore, while these genes were most common among species more closely related to *C. jejuni* (Fig. 1), they were absent in the *C. coli* strain that we examined, which is somewhat surprising, given the close relatedness of these two species and the rate of genetic exchange between them [19], [20], but these results are nonetheless consistent with previous observations in which MeOPN was not detected for any of the 18 *C. coli* isolates we examined [10]. It should be pointed out however, that 3 other sequenced strains of *C. coli*, including the type strain, do contain the *cj1416-cj1418* orthologues, but no obvious *cj1421/cj1422* orthologues. Twelve of the *Campylobacter* species containing orthologous genes were analyzed by HSQC HR-MAS NMR to screen for the presence of MeOPN, and resonances consistent with MeOPN were observed for the majority of *Campylobacter* strains screened (9 of 13). One possible reason why we did not detect MeOPN in the other *Campylobacter* species is that these strains could produce a phosphoramidate lacking the *O*-methyl group, as has been observed in the lipopolysaccharides of *Xanthomonas campestris* [21] and *Shewanella* [22], because the diagnostic signal in the HSQC spectra is based on detection of the protons in the *O*-methyl of the MeOPN group. Alternatively, the MeOPN biosynthesis or transferase genes in these strains could be phased off, given that the MeOPN biosynthesis and transferase genes identified in both *C. jejuni* 11168H and *C. jejuni* 81–176 were found to be phase variable [10]; however, the *cj1416-cj1418* orthologues of *C. fetus* subsp. *fetus*, *C. fetus* subsp. *venerealis*, *C. lari* subsp. *lari*, and *C. sputorum* bv. *sputorum* strains tested in this study do not contain GC tracts typically associated with phase variability in *Campylobacter*. Most likely the reason for not detecting MeOPN in these strains is due to the lack of MeOPN transferase orthologues (*cj1421* or *cj1422*) although it is possible they may contain MeOPN transferase genes with low similarity to *cj1421* or *cj1422.*

To investigate the biological role of MeOPN, we used *C. jejuni* 81–176 due to the virulent nature of this strain. We generated mutants that were deficient in both the biosynthesis and transfer of MeOPN, to be confident that any effects observed could be correlated specifically to loss of MeOPN, and confirmed that there were no obvious changes to CPS besides the lack of MeOPN. The identification of two orthologues of *cj1422*, with one phased off in our 81–176 wild-type straiņ is interesting because it is unclear whether an isolate with both these genes phased on would display a second distinct MeOPN modification or whether the transferases play redundant roles. The published structure of the 81–176 CPS indicates only one MeOPN modification [23], [24], but it is not known whether both transferases are functional in this background. To avoid potential confusion due to phase variation, we generated a mutant with both of these genes disrupted.

We first examined whether or not the presence/absence of MeOPN on the cell surface affects the adherence/invasion and serum resistance of *C. jejuni*. The results obtained from the assays indicated that MeOPN influences the ability of *C. jejuni* to invade and resist serum killing. It is noteworthy that the serum resistance results with the *cjj81176_1420*::*kan^r^*/*cjj81176_1435*::*cam^r^* mutant are consistent with those recently reported by Maue *et al*. [15] with a *C. jejuni* 81–176 MeOPN biosynthetic mutant, which we also tested. Because *C. jejuni* is primarily considered a gastrointestinal pathogen, these results are relevant as they indicate that CPS MeOPN modifications play a role in protection against humoral immunity. However, because *C. jejuni* can also cause bacteremia [25], these observations may be directly relevant to infections in this context. To better understand the mechanisms underlying the improved serum resistance of wild-type 81–176 versus the MeOPN transferase mutant, we examined what effect, if any, the addition of EGTA to serum assays would have on bacterial survival. EGTA is a chelator with high selectivity for Ca^2+^; therefore, the addition of EGTA acts as an effective inhibitor of the classical pathway of complement activation, which is Ca^2+^-dependent [26]. The effect of EGTA addition was drastic in the case of the MeOPN mutant, which was unable to survive a 60 min incubation with serum in the absence of EGTA but showed ∼100% survival when EGTA was added, indicating that the serum killing observed is mediated primarily via the classical complement pathway. This is consistent with our observation that the commercial serum that we used contained *C. jejuni* antibodies. The intermediate level of serum survival observed with the wild-type strain in the absence of EGTA indicates that the CPS MeOPN modifications offer a moderate degree of protection by interfering with serum killing.

The phase variability of the *cjj81176_1420* and *cjj81176_1435* genes suggests that MeOPN expression is desirable early in the infection process for protection against humoral immunity, but that phase variation to a MeOPN^−^ phenotype later in the infection process would be beneficial and enhance the invasiveness and virulence of *C. jejuni*. Interestingly, the *C. jejuni* NCTC 11168 variant that we isolated, which became bacteriophage resistant through the loss of MeOPN [11], also showed increased invasiveness relative to the parental strain (M.C. Holst-Sorensen *et al*., unpublished data). It is not yet clear whether or not the presence of MeOPN enhances serum resistance by preventing antibody deposition, by inhibiting binding by CRP or another acute phase serum protein, or by functioning through another mechanism. Studies are currently underway to determine if CRP plays a role in the observed serum mediated killing and whether other components of human serum bind preferentially to whole cells and/or CPS lacking MeOPN relative to wild-type.

Given that MeOPN was found to influence both serum resistance and invasion, we proceeded to examine the effects of loss of MeOPN *in vivo* in both commensal (chicken) and mammalian (piglet) colonization models. Loss of MeOPN in the 81–176 transferase mutant was found to have no effect on chicken colonization. This is consistent with our recent results demonstrating that *C. jejuni* NCTC 11168 fed to chickens along with bacteriophages recognizing the MeOPN modification caused a selection for *C. jejuni* variants without the CPS modification, but did not change levels of *C. jejuni* colonization [12]. However, MeOPN did influence colonization of piglets relative to wild-type suggesting that this modification may be important in an infection model, but it is still premature to make any definitive conclusions since one model is using direct challenge while the other model used competitive challenge. Combined, these results suggest that MeOPN does not play a significant role in commensal colonization, but may play a contributory role in pathogenesis. Maue *et al*. [15] also recently reported that an 81–176 MeOPN biosynthesis mutant showed a reduction in colonization levels compared to wild-type 10 days post-infection in mice, another mammalian model system. These studies suggest an interplay between the benefits and disadvantages of MeOPN expression at various stages of the infection process. It is interesting to note that, although the presence of MeOPN had an effect on piglet colonization, MeOPN modifications were not observed for any of the 19 *C. coli* strains that we have examined by HR-MAS NMR to date [10], in spite of the latter having a greater affinity for pigs as a host [27]. It will be interesting to explore these relationships further and to examine a broader range of isolates within each species [20]. It is also possible that the primary role of MeOPN modification may in fact be in the mediation of interactions between *Campylobacter* species and phages and we have already demonstrated that this relationship indeed exists for *C. jejuni* [11], [12].

In a previous study investigating the ability of *C. jejuni* 11168H to kill *G. mellonella* larvae, a MeOPN biosynthetic mutant showed a marked decrease in larval killing relative to wild-type and complementation of the mutation was able to restore wild-type killing, suggesting that MeOPN has insecticidal activity against *G. mellonella* [14]. Given that MeOPN appeared to be responsible for the larval killing observed, we were interested in testing MeOPN transferase mutants in the larval killing assay. The ability of both the *C. jejuni* 81–176 and *C. jejuni* 11168H MeOPN transferase mutants to kill *G. mellonella* larvae to wild-type levels was unexpected, and suggested that MeOPN might be accumulating to significant levels within the bacterial cell in our mutants. However, we were unable to detect MeOPN or MeOPN intermediates in metabolomics studies with the *cjj81176_1420*::*kan^r^*/*cjj81176_1435*::*cam^r^* mutant (results not shown), so we examined potential insecticidal activity directly. MeOPN-linked monosaccharides were synthesized and injected into *G. mellonella* larvae, but none of the compounds showed any level of insecticidal activity. To rule out the possibility that MeOPN needs to be presented in a specific context to elicit the insecticidal activity, purified CPS from 81–176 wild-type was injected into *G. mellonella*, but again no insecticidal activity was observed. It is worth noting that concentrated solutions were used for both the MeOPN-linked monosaccharides and the purified CPS injections, and therefore the MeOPN levels in these injections are significantly higher than those in the *C. jejuni* injections. The inability of MeOPN, MeOPN-linked oligosaccharides, or purified 81–176 CPS to induce larval killing provided conclusive evidence that MeOPN does not have insecticidal activity as previously suggested [14].

While it is possible that MeOPN could contribute indirectly to larval killing by protecting the bacteria from the humoral responses of the larval immune system, the ability of the *cjj81176_1420*::*kan^r^*/*cjj81176_1435*::*cam^r^* mutant to kill larvae at wild-type levels suggests that larval killing by *C. jejuni* is MeOPN-independent. The observation that MeOPN biosynthetic mutants show a decreased ability to kill *G. mellonella* larvae as observed by Champion *et al*. and by us in this study, is contrary to the other findings, and suggests that MeOPN biosynthetic intermediates may accumulate in the cytoplasm, causing the mutant to be less able to survive inside the larvae, or that the mutation causes downstream effects. Examination of all the mutants in this study by RT-PCR demonstrated wild-type levels of gene transcripts downstream of both MeOPN transferase mutants. In contrast, mutants in the biosynthesis homologue *cj1416* (which is also the gene mutated by Champion *et al*.) show slightly less *cj1415* transcription in both strain backgrounds. This is of relevance because in our earlier study [10], we noted that *cj1415* mutation results in reduced CPS expression (based on the need to do 2816 NMR scans of the *cj1415* mutant to see similar capsule resonance signals compared to 256 scans for the wild-type and other MeOPN mutants). So, the MeOPN biosynthesis mutants may show higher levels of survival compared to the wild-types because they are actually producing less CPS. Future studies to elucidate the biochemical pathway for MeOPN biosynthesis and to develop methods to accurately quantitate CPS expression will be invaluable to advance our understanding of this unique modification.

In summary, we have demonstrated that MeOPN modifications are common within the *Campylobacter* genus, and especially prevalent in species more closely related to *C. jejuni*, with the possible exception of *C. coli*. We identified MeOPN biosynthesis and transferase genes in *C. jejuni* 81–176, and generated mutants in this pathway. We demonstrated that MeOPN moieties are not inherently insecticidal against *G. mellonella* and that larval killing by *C. jejuni* is not directly related to MeOPN. We further showed that loss of MeOPN on the cell surface results in a drastic decrease in serum resistance while enhancing invasion of Caco-2 cells. Loss of MeOPN was found to have no effect on colonization in a commensal chicken model, but showed a reduction in colonization relative to wild-type in a piglet model, suggesting that MeOPN has a contributory role in pathogenesis.

## Supporting Information

Figure S1 **RT-PCR analyses of genes located downstream from the** ***Campylobacter jejuni*** **mutated genes in this study.** Reverse Transcriptase PCR analyses of genes downstream of the chromosomally disrupted genes indicates similar transcript levels between wild-type and MeOPN transferase mutants for (A) *cj1420* downstream of *cj1421* and *cj1422* in *C. jejuni* 11168H and *cjj81176_1419* downstream of *cjj81176_1420* in *C. jejuni* 81–176, and (B) *cjj81176_1434* downstream of *cjj81176_1435* in the 81–176 background. Lower levels of transcripts were observed in the MeOPN biosynthesis mutant backgrounds relative to the wild-type strain for (C) *cj1415* downstream of *cj1416* in 11168H and *cjj81176_1414* downstream of *cjj81176_1415* in 81–176.
(DOC)

Table S1 **Accession numbers for the Cj1416-Cj1418 homologues and AtpA sequences from** ***Campylobacter*** **species used in this study.**
(DOC)
