# Supplementary material for: Biological Roles of the O-Methyl Phosphoramidate Capsule Modification in Campylobacter jejuni
Source: PLoS One. 2014 Jan 30;9(1):e87051. doi: 10.1371/journal.pone.0087051 (PMC3907429; doi:10.1371/journal.pone.0087051)
Supplement: Table S1 — Accession numbers for the Cj1416-Cj1418 homologues and AtpA sequences from Campylobacter species used in this study. (DOC) [file pone.0087051.s002.doc]

| BankIt1674805 Seq4 | KF855248 | >Seq4 [organism=Campylobacter lari] [sub_species=concheus] [strain=LMG 11760] phosphoramidate cytidylyltransferase | Cj1416 |
| --- | --- | --- | --- |
| BankIt1674805 Seq3 | KF855249 | >Seq3 [organism=Campylobacter insulaenigrae] [strain=NCTC 12927] phosphoramidate cytidylyltransferase | Cj1416 |
| BankIt1674805 Seq5 | KF855250 | >Seq5 [organism=Campylobacter subantarcticus] [strain=LMG 24377] phosphoramidate cytidylyltransferase | Cj1416 |
| BankIt1674805 Seq7 | KF855251 | >Seq7 [organism=Campylobacter upsaliensis] [strain=RM3195] phosphoramidate cytidylyltransferase | Cj1416 |
| BankIt1674805 Seq1 | KF855252 | >Seq1 [organism=Campylobacter cuniculorum] [strain=LMG 24588] phosphoramidate cytidylyltransferase | Cj1416 |
| BankIt1674805 Seq2 | KF855253 | >Seq2 [organism=Campylobacter helveticus] [strain=ATCC 51209] phosphoramidate cytidylyltransferase | Cj1416 |
| BankIt1674805 Seq9 | KF855254 | >Seq9 [organism=Campylobacter sputorum] [biovar=sputorum] [strain=RM3237] phosphoramidate cytidylyltransferase | Cj1416 |
| BankIt1674805 Seq6 | KF855255 | >Seq6 [organism=Campylobacter upsaliensis] [strain=RM3940] phosphoramidate cytidylyltransferase | Cj1416 |
| BankIt1674805 Seq8 | KF855256 | >Seq8 [organism=Campylobacter lari] [type=UPTC] [strain=NCTC 11845] phosphoramidate cytidylyltransferase | Cj1416 |
|  |  |  |  |
| BankIt1674805 Seq13 | KF855257 | >Seq13 [organism=Campylobacter lari] [sub_species=concheus] [strain=LMG 11760] type I glutamine amidotransferase | Cj1417 |
| BankIt1674805 Seq12 | KF855258 | >Seq12 [organism=Campylobacter insulaenigrae] [strain=NCTC 12927] type I glutamine amidotransferase | Cj1417 |
| BankIt1674805 Seq14 | KF855259 | >Seq14 [organism=Campylobacter subantarcticus] [strain=LMG 24377] type I glutamine amidotransferase | Cj1417 |
| BankIt1674805 Seq16 | KF855260 | >Seq16 [organism=Campylobacter upsaliensis] [strain=RM3195] type I glutamine amidotransferase | Cj1417 |
| BankIt1674805 Seq10 | KF855261 | >Seq10 [organism=Campylobacter cuniculorum] [strain=LMG 24588] type I glutamine amidotransferase | Cj1417 |
| BankIt1674805 Seq11 | KF855262 | >Seq11 [organism=Campylobacter helveticus] [strain=ATCC 51209] type I glutamine amidotransferase | Cj1417 |
| BankIt1674805 Seq18 | KF855263 | >Seq18 [organism=Campylobacter sputorum] [biovar=sputorum] [strain=RM3237] type I glutamine amidotransferase | Cj1417 |
| BankIt1674805 Seq15 | KF855264 | >Seq15 [organism=Campylobacter upsaliensis] [strain=RM3940] type I glutamine amidotransferase | Cj1417 |
| BankIt1674805 Seq17 | KF855265 | >Seq17 [organism=Campylobacter lari] [type=UPTC] [strain=NCTC 11845] type I glutamine amidotransferase | Cj1417 |
|  |  |  |  |
| BankIt1674805 Seq22 | KF855266 | >Seq22 [organism=Campylobacter lari] [sub_species=concheus] [strain=LMG 11760] phosphate kinase | Cj1418 |
| BankIt1674805 Seq21 | KF855267 | >Seq21 [organism=Campylobacter insulaenigrae] [strain=NCTC 12927] phosphate kinase | Cj1418 |
| BankIt1674805 Seq23 | KF855268 | >Seq23 [organism=Campylobacter subantarcticus] [strain=LMG 24377] phosphate kinase | Cj1418 |
| BankIt1674805 Seq25 | KF855269 | >Seq25 [organism=Campylobacter upsaliensis] [strain=RM3195] phosphate kinase | Cj1418 |
| BankIt1674805 Seq19 | KF855270 | >Seq19 [organism=Campylobacter cuniculorum] [strain=LMG 24588] phosphate kinase | Cj1418 |
| BankIt1674805 Seq20 | KF855271 | >Seq20 [organism=Campylobacter helveticus] [strain=ATCC 51209] phosphate kinase | Cj1418 |
| BankIt1674805 Seq27 | KF855272 | >Seq27 [organism=Campylobacter sputorum] [biovar=sputorum] [strain=RM3237] phosphate kinase | Cj1418 |
| BankIt1674805 Seq24 | KF855273 | >Seq24 [organism=Campylobacter upsaliensis] [strain=RM3940] phosphate kinase | Cj1418 |
| BankIt1674805 Seq26 | KF855274 | >Seq26 [organism=Campylobacter lari] [type=UPTC] [strain=NCTC 11845] phosphate kinase | Cj1418 |
|  |  |  |  |
| BankIt1674828 Seq1 | KF855275 | >Seq1 [organism=Campylobacter avium] [strain=LMG 24591] | atpA |
| BankIt1674828 Seq2 | KF855276 | >Seq2 [organism=Campylobacter canadensis] [strain=L266] | atpA |
| BankIt1674828 Seq3 | KF855277 | >Seq3 [organism=Campylobacter coli] [strain=RM2228] | atpA |
| BankIt1674828 Seq4 | KF855278 | >Seq4 [organism=Campylobacter cuniculorum] [strain=LMG 24588] | atpA |
| BankIt1674828 Seq5 | KF855279 | >Seq5 [organism=Campylobacter fetus] [sub_species=fetus] [strain=82-40] | atpA |
| BankIt1674828 Seq6 | KF855280 | >Seq6 [organism=Campylobacter fetus] [sub_species=venerealis] [strain=NCTC 10354] | atpA |
| BankIt1674828 Seq7 | KF855281 | >Seq7 [organism=Campylobacter gracilis] [strain=ATCC 33236] | atpA |
| BankIt1674828 Seq8 | KF855282 | >Seq8 [organism=Campylobacter helveticus] [strain=ATCC 51209] | atpA |
| BankIt1674828 Seq9 | KF855283 | >Seq9 [organism=Campylobacter hyointestinalis] [sub_species=hyointestinalis] [strain=LMG 9260] | atpA |
| BankIt1674828 Seq10 | KF855284 | >Seq10 [organism=Campylobacter hyointestinalis] [sub_species=lawsonii] [strain=CCUG 27631] | atpA |
| BankIt1674828 Seq11 | KF855285 | >Seq11 [organism=Campylobacter hominis] [strain=LMG 19568] | atpA |
| BankIt1674828 Seq12 | KF855286 | >Seq12 [organism=Campylobacter insulaenigrae] [strain=NCTC 12927] | atpA |
| BankIt1674828 Seq13 | KF855287 | >Seq13 [organism=Campylobacter jejuni] [sub_species=doylei] [strain=269.97] | atpA |
| BankIt1674828 Seq14 | KF855288 | >Seq14 [organism=Campylobacter jejuni] [sub_species=jejuni] [strain=RM1221] | atpA |
| BankIt1674828 Seq15 | KF855289 | >Seq15 [organism=Campylobacter lanienae] [strain=NCTC 13004] | atpA |
| BankIt1674828 Seq16 | KF855290 | >Seq16 [organism=Campylobacter lari] [sub_species=lari] [strain=RM2100] | atpA |
| BankIt1674828 Seq17 | KF855291 | >Seq17 [organism=Campylobacter lari] [sub_species=concheus] [strain=LMG 11760] | atpA |
| BankIt1674828 Seq18 | KF855292 | >Seq18 [organism=Campylobacter mucosalis] [strain=CCUG 21559] | atpA |
| BankIt1674828 Seq19 | KF855293 | >Seq19 [organism=Campylobacter concisus] [strain=13826] | atpA |
| BankIt1674828 Seq20 | KF855294 | >Seq20 [organism=Campylobacter peloridis] [strain=LMG 23910] | atpA |
| BankIt1674828 Seq21 | KF855295 | >Seq21 [organism=Campylobacter rectus] [strain=ATCC 33238] | atpA |
| BankIt1674828 Seq22 | KF855296 | >Seq22 [organism=Campylobacter sputorum] [biovar=fecalis] [strain=CCUG 20703] | atpA |
| BankIt1674828 Seq23 | KF855297 | >Seq23 [organism=Campylobacter showae] [strain=ATCC 51146] | atpA |
| BankIt1674828 Seq24 | KF855298 | >Seq24 [organism=Campylobacter sputorum] [biovar=paraureolyticus] [strain=LMG 11764] | atpA |
| BankIt1674828 Seq25 | KF855299 | >Seq25 [organism=Campylobacter sputorum] [biovar=sputorum] [strain=RM3237] | atpA |
| BankIt1674828 Seq26 | KF855300 | >Seq26 [organism=Campylobacter subantarcticus] [strain=LMG 24377] | atpA |
| BankIt1674828 Seq27 | KF855301 | >Seq27 [organism=Campylobacter upsaliensis] [strain=RM3940] | atpA |
| BankIt1674828 Seq28 | KF855302 | >Seq28 [organism=Campylobacter ureolyticus] [strain=RIGS 9880] | atpA |
| BankIt1674828 Seq29 | KF855303 | >Seq29 [organism=Campylobacter curvus] [strain=525.92] | atpA |
| BankIt1674828 Seq30 | KF855304 | >Seq30 [organism=Campylobacter volucris] [strain=LMG 24379] | atpA |
| BankIt1674828 Seq31 | KF855305 | >Seq31 [organism=Campylobacter lari] [type=UPTC] [strain=NCTC 11845] | atpA |
