# Supplementary material for: Biological Roles of the O-Methyl Phosphoramidate Capsule Modification in Campylobacter jejuni
Source: PLoS One. 2014 Jan 30;9(1):e87051. doi: 10.1371/journal.pone.0087051 (PMC3907429; doi:10.1371/journal.pone.0087051)
Supplement: Supplementary file 2 [file pone.0087051.s001.doc]

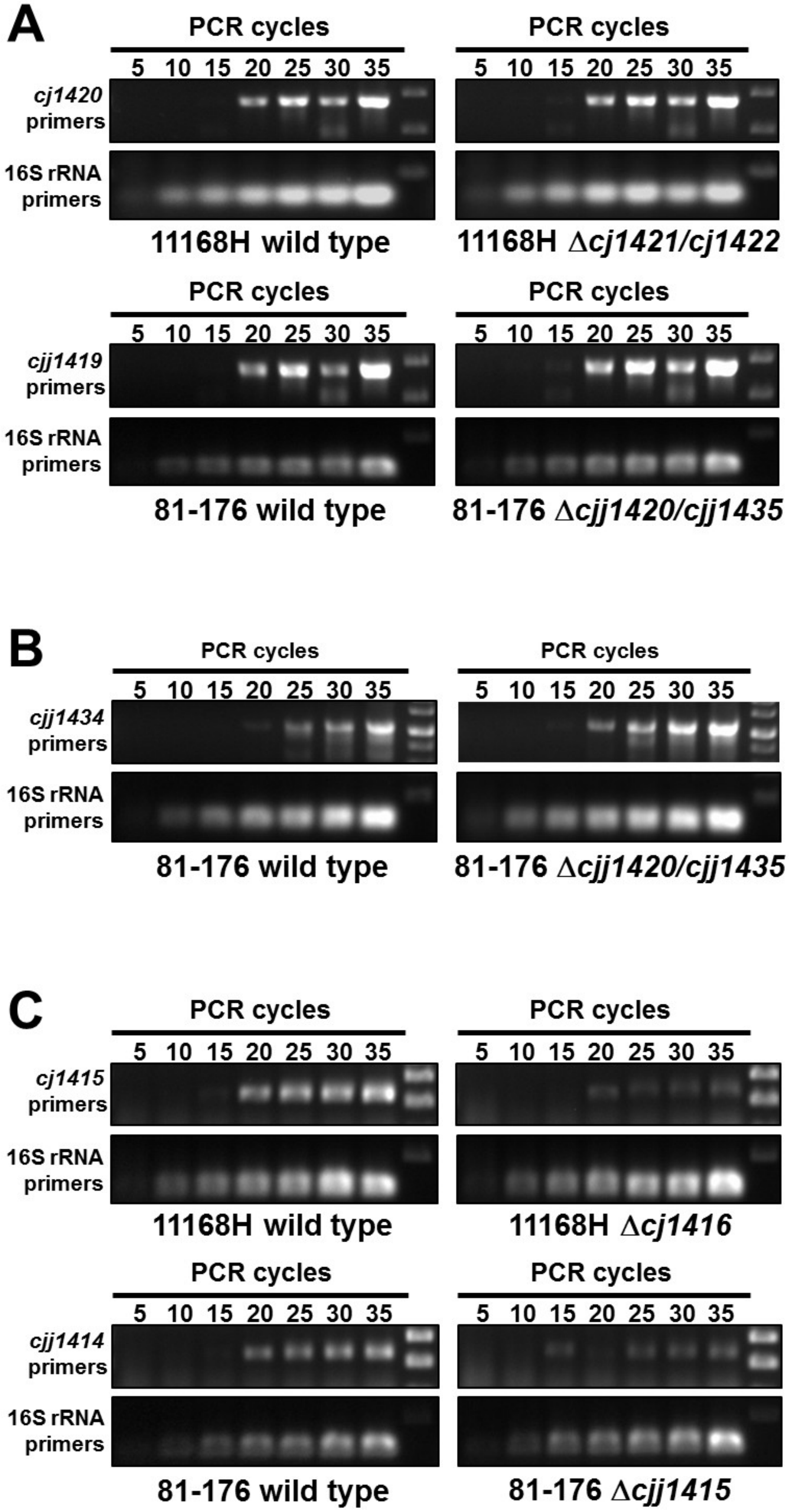
A
PCR cycles
5 10 15 20 25 30 35
cj1420 primers
16S rRNA primers
11168H wild type
11168H Δcj1421/cj1422
cjj1419 primers
81-176 wild type
81-176 Δcjj1420/cjj1435
B
cjj1434 primers
C
cj1415 primers
11168H Δcj1416
cjj1414 primers
81-176 Δcjj1415
